# The endothelial activation and stress index as a key prognostic marker in severe ischemic stroke

**DOI:** 10.1186/s40001-025-03823-6

**Published:** 2026-01-09

**Authors:** Rui-Yun Yu, Zhi-Na Liu, Yue Wang, Tao Wang

**Affiliations:** 1 Xinxiang Central Hospital, Xinxiang, China; 2 The Fourth Clinical College of Xinxiang Medical University, Xinxiang, China; 3 The Affiliated Hospital of Yan’an University, Yan’an, China

**Keywords:** EASIX, Ischemic stroke, All-cause mortality, Endothelial dysfunction, MIMIC-IV

## Abstract

**Background:**

Severe ischemic stroke (SIS) is a life-threatening condition associated with high mortality rates. Endothelial dysfunction plays a critical role in its progression, leading to further neurovascular damage. The endothelial activation and stress index (EASIX) is a simple and easily accessible biomarker and may serve as a potential prognostic indicator for SIS. This study investigates the relationship between EASIX levels and 28-day all-cause mortality (ACM) in patients with SIS.

**Methods:**

The study utilized data from the Medical Information Mart for Intensive Care-IV (MIMIC-IV) database. Patients were stratified into three cohorts based on log_2_-transformed EASIX values. The association between EASIX and 28-day ACM in SIS was evaluated using Cox proportional hazards models, Kaplan–Meier survival analysis, restricted cubic spline (RCS) regression, and subgroup analyses. Receiver operating characteristic (ROC) curve analysis was performed to compare the predictive accuracy of EASIX with other prognostic markers.

**Results:**

A total of 786 patients with SIS were included in the study. The patients were stratified into tertiles based on their log₂(EASIX) values. Elevated EASIX levels were associated with prolonged hospital and ICU stays as well as an increased risk of 28-day ACM. Kaplan–Meier survival analysis revealed that higher EASIX levels significantly correlated with reduced survival probability. Cox regression analysis indicated that each unit increase in log₂(EASIX) was associated with a 17% higher mortality risk. Moreover, patients in the highest EASIX tertile exhibited a significantly greater mortality risk. RCS regression further identified a nonlinear increase in mortality risk beyond an EASIX threshold of 0.58. ROC analysis demonstrated that log₂(EASIX) had superior predictive accuracy for 28-day ACM (AUC = 0.765), outperforming both the SOFA and SIRS scores. Subgroup analyses confirmed the robustness of this association across various patient characteristics, with no significant interactions.

**Conclusion:**

EASIX is a simple and accessible biomarker that provides early warning for identifying high-risk patients. Elevated EASIX levels are strongly associated with an increased risk of 28-day ACM in SIS patients.

**Graphical Abstract:**

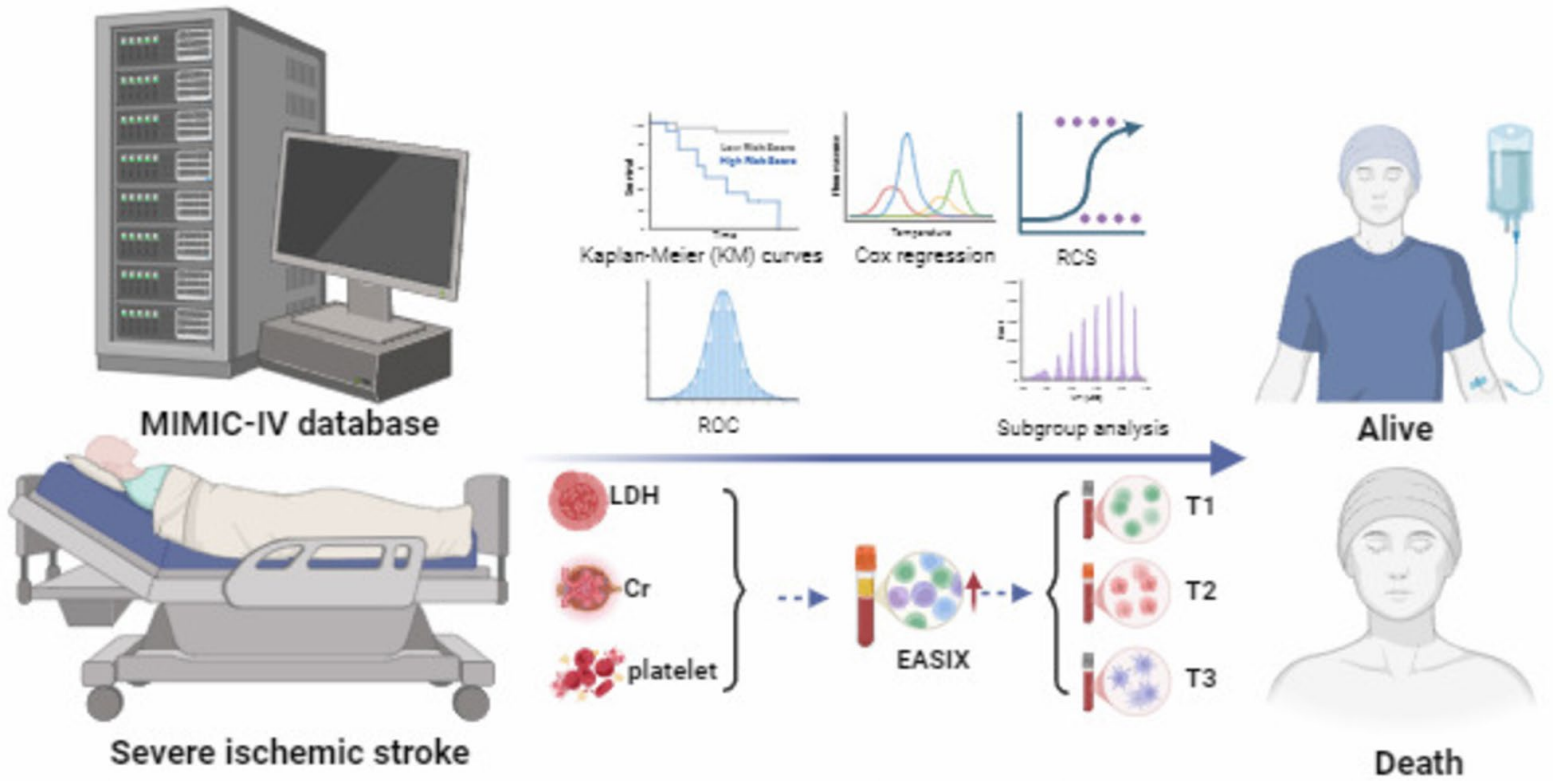

## Introduction

According to the Global Burden of Disease (GBD) Study 2021, ischemic stroke continues to impose a significant global burden, with a notable rise in the number of cases [1]. As the second leading cause of death worldwide, it accounts for approximately 5.9 million deaths annually [2, 3]. Severe ischemic stroke (SIS), one of the most devastating subtypes, represents 20–30% of all ischemic strokes [4] and is associated with exceptionally high mortality and disability rates. Studies indicate that the 28-day all-cause mortality (ACM) among SIS patients ranges from 25 to 40%, with over 60% of survivors experiencing persistent neurological impairments of varying severity [5]. SIS is one of the most prevalent and life-threatening conditions in the neurocritical care unit (NICU), contributing significantly to in-hospital mortality. Therefore, the early identification of biomarkers predictive of SIS-related mortality is essential for improving clinical management, reducing mortality, and enhancing patient outcomes [6].

Endothelial dysfunction plays a critical role in the pathogenesis of ischemic stroke, exacerbating neurovascular damage through the activation of inflammatory cascades, disruption of microcirculatory homeostasis, imbalance in vasoactive mediators, and induction of endothelial apoptosis. These processes ultimately lead to blood–brain barrier (BBB) disruption and neuronal injury [7–9], worsening neurological impairment and potentially resulting in fatal outcomes. While EASIX has shown prognostic value in hematologic and septic contexts, its relevance in neurovascular conditions like SIS remains unexplored. EASIX, a novel biomarker indicative of endothelial dysfunction, has gained recognition in critically ill patients, yet its potential as a prognostic marker in SIS has not been fully evaluated [10–13]. Based on this existing evidence, we hypothesize that EASIX could be a valuable tool for prognostic assessment in SIS.

The Medical Information Mart for Intensive Care-IV database is a large, publicly accessible repository of clinical data, offering a rich source of real-world information from critically ill patients. In this study, we utilized the MIMIC-IV database to assess the association between EASIX and 28-day ACM in SIS patients. By evaluating the predictive utility of EASIX, we aim to develop a novel tool for early risk stratification in critically ill stroke patients, refine NICU management strategies, and ultimately improve the prognosis of SIS.

## Methods

### Data source

The data for this study were obtained from the MIMIC-IV (v2.2) database, an open-access medical repository curated by the Laboratory for Computational Physiology at the Massachusetts Institute of Technology (MIT-LCP) [14]. To ensure patient confidentiality, all data underwent strict de-identification procedures, with personally identifiable information replaced by randomly generated codes. Our research team has met all necessary training requirements and completed the "Conflict of Interest" and "Research Data or Specimen Only" certifications, securing authorized access to the dataset.

### Study population

**Fig. 1:**
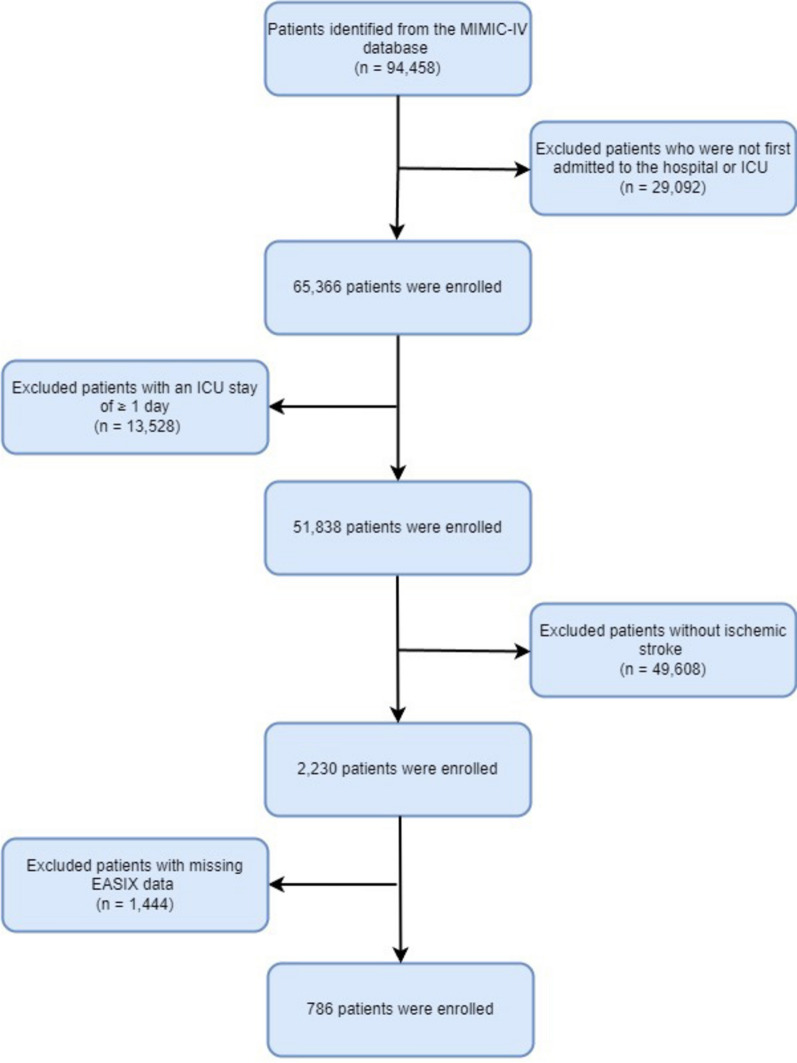
Flowchart of the participants’ selection from the MIMIC-IV database

The MIMIC-IV database was utilized to identify patients admitted between 2008 and 2019 who met the following inclusion criteria: adults (≥ 18 years) diagnosed with ischemic stroke, classified using ICD-9 codes 433/434/436/437.0/437.1 or ICD-10 codes I63/I65/I66. For patients with multiple ICU admissions, only the first admission record was included in the analysis. The exclusion criteria were: (i) incomplete EASIX-related data; (ii) ICU length of stay < 24 h; (iii) missing data exceeding 10%. For the remaining missing data, multiple imputations were performed using the Fully Conditional Specification (FCS) method. After applying these selection criteria and imputing missing data, a final cohort of 786 patients was included for analysis (Fig. 1).

### Data preparation and definitions

The EASIX score was calculated as follows: EASIX = LDH (U/L) × creatinine (mg/dL)/platelet count (10⁹/L). Previous studies have shown that the EASIX score follows a right-skewed distribution, necessitating a log₂ transformation for normalization. To facilitate subsequent analysis, EASIX values were stratified into tertiles based on their distribution [15]. This tertile stratification, commonly employed in similar studies, enables the identification of threshold effects and improves the interpretation of risk across different levels of the biomarker [16].

The covariates were grouped into five primary categories: (i) demographic characteristics (e.g., age, sex); (ii) vital signs, including mean arterial pressure (MAP), temperature, respiratory rate, and oxygen saturation (SpO₂); (iii) laboratory biomarkers, encompassing blood biochemistry, complete blood count, and coagulation parameters; (iv) scoring systems, such as the Sequential Organ Failure Assessment (SOFA), Acute Physiology Score III (APS III), Glasgow Coma Scale (GCS), Charlson Comorbidity Index (CCI), and Systemic Inflammatory Response Syndrome (SIRS); (v) clinical treatment data, including thrombolysis and thrombectomy; and (vi) comorbidities, such as diabetes, hypertension, malignancy, and renal failure. To ensure data integrity and analytical consistency, rigorous quality control measures were implemented throughout the study, including independent verification of key variables, consistency assessments, and automated error detection and correction using statistical software.

### Study outcomes

The primary endpoint of this study was 28-day ACM following hospital admission.

### Statistical analysis

Categorical variables were presented as percentages (%) and analyzed using the Chi-square test or Fisher’s exact test. Continuous variables were summarized based on their distributional properties: normally distributed variables were reported as mean ± SD and compared using the independent t-test, whereas skewed variables were presented as the median and interquartile range (IQR) and analyzed using the Mann–Whitney U test or Kruskal–Wallis test.

Kaplan–Meier survival analysis was performed to assess differences in the distribution of 28-day ACM among patients with SIS stratified by EASIX levels. Cox proportional hazards regression models were employed to investigate the association between log₂(EASIX) and 28-day ACM, estimating hazard ratios (HRs) with corresponding 95% confidence intervals (CIs). To account for potential confounders, three adjustment models were constructed: (i) crude model (unadjusted for covariates); (ii) Model 1 (adjusted for age, sex, GCS, and CCI); and (iii) Model 2 (additionally adjusted for heart failure, respiratory failure, atrial fibrillation, diabetes, hypertension, hyperlipidemia, renal failure, malignancy, MAP, temperature, SpO₂, respiratory rate, heart rate, intravenous thrombolysis, and thrombectomy). Patients were stratified into tertiles according to EASIX levels, with the lowest tertile (T1) serving as the reference group, and adjusted HRs for 28-day ACM estimated for the remaining tertiles. Furthermore, restricted cubic spline (RCS) regression was performed to examine the nonlinear association between log₂(EASIX) and 28-day ACM.

All statistical analyses were conducted using R software (version 4.3.1; R Foundation for Statistical Computing, Vienna, Austria) with the following packages: survival, rms, pROC, and ggplot2.

## Results

### Baseline characteristics

A total of 786 patients were included in this study. The median hospital stay was 9.6 days, and the median ICU stay was 4.16 days. The overall 28-day ACM rate was 17%. Patients were divided into three groups based on EASIX levels: T1 (low: −3.77 to −0.07), T2 (moderate: −0.07 to 1.28), and T3 (high: 1.28 to 7.80). Patients in the T3 group had significantly longer hospital stays (15.85 days) and ICU stays (5.44 days) compared to the T1 group. The 28-day ACM rate was also higher in T3 (P < 0.01). The T3 group had a higher incidence of respiratory failure and renal failure, with rates of 61% and 70%, respectively, compared to 19% and 9% in the T1 group. Additionally, hypertension and atrial fibrillation were more prevalent in the T3 group. The SOFA score was significantly higher in T3 (7) compared to T1 (2), and APS III scores were also higher in T3 (55 vs. 32). Laboratory findings indicated that patients in T3 had significantly lower red blood cell counts, hemoglobin levels, and higher blood urea nitrogen levels compared to the T1 group. Detailed results are summarized in Table 1.

**Table 1:** Baseline characteristics of participants

Characteristic	Overall, *N* = 786 (100%)	T1 *N* = 263 (33%)	T2 *N* = 261 (33%)	T3 *N* = 262 (33%) (33%)^1^	*P* value
EASIX	−0.69 [−3.76, −0.07]	0.58(−0.074,1.281]	0.58 (0.23, 0.93)	2.60 (1.28,7.80]	** < 0.001**
Demographics					
Age (years)	70 (59, 80)	67 (55, 79)	73 (64, 84)	68 (57, 80)	** < 0.001**
Sex					** < 0.001**
Female	394 (50%)	162 (62%)	112 (43%)	120 (46%)	
Male	392 (50%)	101 (38%)	149 (57%)	142 (54%)	
Vital signs					
MAP (mmHg)	86.5 (78.5, 96.3)	89.7 (80.8, 101.1)	88.4 (79.8, 97.6)	82.5 (74.8, 89.3)	** < 0.001**
Temperature (°C)	36.9 (36.7, 37.1)	36.9 (36.7, 37.1)	36.9 (36.7, 37.1)	36.9 (36.7, 37.3)	0.6
Respiratory rate (times/min)	19 (17, 22)	19 (17, 21)	19 (17, 21)	21 (18, 24)	** < 0.001**
SpO2 (%)	97 (96, 98)	97 (96, 98)	97 (96, 98)	97 (96, 99)	> 0.9
Laboratory-based data					
RBC 10^12^/L	3.82 (3.22, 4.30)	4.09 (3.64, 4.41)	3.90 (3.38, 4.38)	3.32 (2.79, 4.02)	** < 0.001**
WBC, 10^9^/L	9.2 (7.1, 12.4)	9.0 (7.3, 11.3)	8.9 (7.0, 12.2)	9.7 (7.0, 14.1)	0.10
PLT, 10^9^/L	213 (166, 279)	269 (225, 325)	199 (176, 260)	163 (108, 219)	** < 0.001**
Hb (g/dL)	11.1 (9.0, 12.9)	11.8 (10.2, 13.2)	11.5 (9.4, 13.2)	9.7 (7.7, 11.6)	** < 0.001**
HCT	34.3 (28.1, 38.9)	36.2 (32.6, 39.7)	35.1 (29.7, 40.2)	29.8 (24.4, 35.6)	** < 0.001**
NEUT 10^9^/L	7.81 (5.55, 10.90)	7.21 (5.45, 9.47)	7.65 (5.49, 10.36)	9.34 (5.83, 13.73)	** < 0.001**
AG (mmol/L)	13 (11, 15)	13 (11, 15)	13 (11, 15)	13 (11, 16)	0.6
BUN (mg/dL)	17 (12, 26)	13 (10, 17)	17 (12, 25)	27 (17, 47)	** < 0.001**
Creatinine (mg/dL)	1.1 (0.8, 1.6)	0.8 (0.7, 0.9)	1.1 (0.9, 1.4)	1.7 (1.2, 2.8)	** < 0.001**
LDH (U/L)	262 (193, 426)	190 (165, 228)	254 (202, 346)	515 (341, 941)	** < 0.001**
GLU (mg/dL)	111 (94, 140)	109 (93, 136)	111 (97, 135)	113 (93, 153)	0.5
Ca (mg/dL)	8.5 (7.9, 9.0)	8.8 (8.4, 9.1)	8.6 (8.1, 9.0)	8.0 (7.4, 8.6)	** < 0.001**
Na (mmol/L)	138 (135, 140)	138 (136, 140)	139 (136, 141)	137 (134, 140)	** < 0.001**
K (mmol/L)	3.9 (3.6, 4.2)	3.9 (3.6, 4.1)	3.9 (3.6, 4.3)	4.0 (3.5, 4.4)	0.091
INR	1.1 (1.1, 1.3)	1.1 (1.0, 1.2)	1.1 (1.1, 1.3)	1.3 (1.1, 1.5)	** < 0.001**
PT (s)	12.4 (11.4, 14.2)	11.8 (11.1, 12.5)	12.4 (11.4, 13.9)	13.7 (12.1, 16.3)	** < 0.001**
ALT (U/L)	19 (12, 35)	16 (10, 23)	18 (13, 29)	31 (16, 122)	** < 0.001**
AST (U/L)	24 (18, 45)	19 (15, 22)	25 (19, 37)	54 (26, 167)	** < 0.001**
Scoring system					
SOFA	4 (2, 7)	2 (1, 3)	3 (2, 5)	7 (5, 10)	** < 0.001**
APS III	40 (30, 54)	32 (24, 43)	38 (29, 49)	55 (43, 74)	** < 0.001**
GCS	14 (11, 15)	14 (12, 15)	14 (11, 15)	15 (11, 15)	0.11
CCI	7 (5, 8)	6 (4, 8)	7 (5, 9)	7 (5, 9)	** < 0.001**
sirs	2 (2, 3)	2 (1, 3)	2 (2, 3)	3 (2, 3)	** < 0.001**
Comorbidities, *n* (%)					
Respiratory failure	295 (38%)	49 (19%)	86 (33%)	160 (61%)	** < 0.001**
Atrial fibrillation	307 (39%)	84 (32%)	117 (45%)	106 (40%)	**0.009**
Diabetes mellitus	279 (35%)	88 (33%)	87 (33%)	104 (40%)	0.2
Malignancy	150 (19%)	52 (20%)	48 (18%)	50 (19%)	> 0.9
Hypertension	594 (76%)	195 (74%)	212 (81%)	187 (71%)	**0.024**
Hyperlipidemia	380 (48%)	133 (51%)	142 (54%)	105 (40%)	**0.003**
Renal failure	291 (37%)	24 (9%)	83 (32%)	184 (70%)	** < 0.001**
Treatment information, *n* (%)					
rt-PA	143 (18%)	30 (11%)	34 (13%)	79 (30%)	** < 0.001**
Thrombectomy	36 (5%)	20 (8%)	14 (5%)	2 (1%)	** < 0.001**
Clinical outcomes					
Hospital stay (days)	9.60 (4.93, 19.08)	7.77 (4.00, 15.02)	8.75 (4.92, 16.45)	15.85 (7.17, 28.70)	** < 0.001**
ICU stay (days)	4.16 (2.24, 8.11)	3.24 (1.94, 6.61)	4.04 (2.09, 7.78)	5.44 (2.97, 10.61)	** < 0.001**
28-day mortality	133 (17%)	14 (5%)	36 (14%)	83 (32%)	** < 0.001**

Bold values indicates statistical significance (*p* <0.05).

### Kaplan–Meier survival analysis

**Fig. 2:**
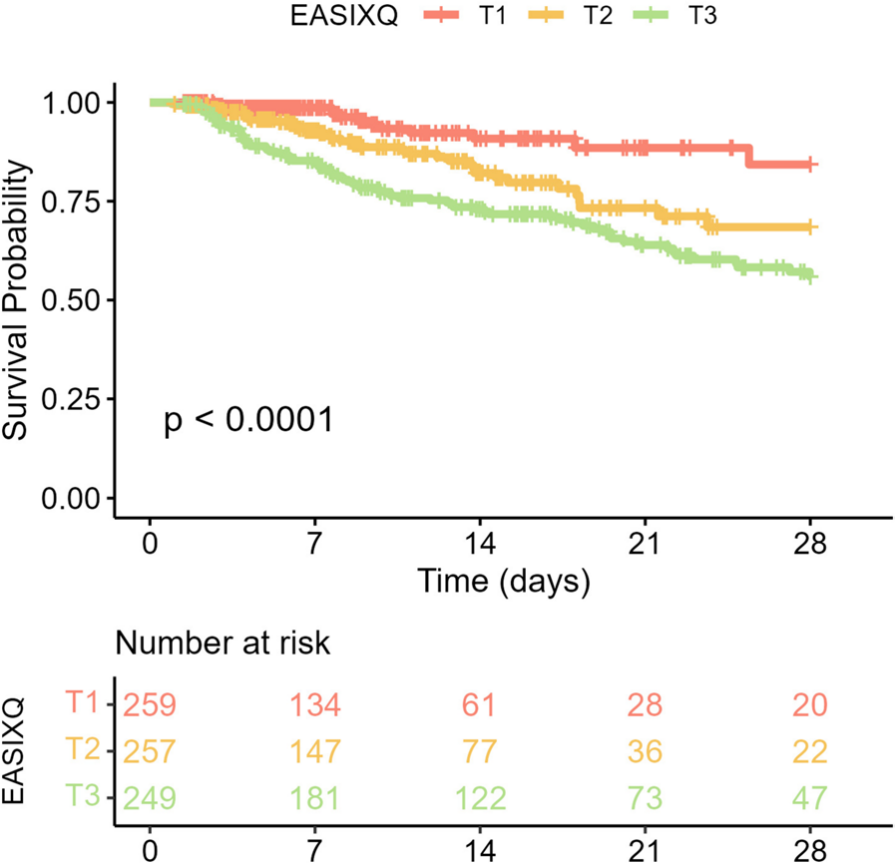
Kaplan–Meier survival analysis curves for 28-day ACM

To explore the relationship between log_2_(EASIX) and 28-day ACM in patients with SIS, Kaplan–Meier survival analysis was conducted. The analysis revealed a significant association between higher EASIX levels and a decreased survival probability (log-rank test, *P* < 0.0001). Among the EASIX tertiles, T3 exhibited the lowest survival probability, followed by T2, while T1 had the highest survival probability (Fig. 2).

### Association between log₂(EASIX) and 28-day ACM in SIS

In the Cox proportional hazards regression analysis examining the impact of EASIX on 28-day ACM in SIS patients, each one-unit increase in log₂(EASIX) was associated with a 17% higher mortality risk in the unadjusted model (HR = 1.17, *p* < 0.0001). After adjusting for age, sex, GCS, and CCI in Model 1, EASIX remained a significant predictor (HR = 1.19, *p* < 0.0001). Further adjustment for clinical variables—including heart failure, respiratory failure, atrial fibrillation, diabetes, hypertension, hyperlipidemia, renal failure, and malignancy—in Model 2 still showed EASIX to be independently associated with increased mortality risk (HR = 1.11, *p* < 0.01).

Additionally, a categorical analysis stratified patients into EASIX tertiles, with T1 as the reference group. In the unadjusted model, the T2 group exhibited a hazard ratio (HR) of 2.19 (*p* = 0.02), while the T3 group had a markedly higher HR of 3.56 (*p* < 0.0001), indicating a significant increase in mortality risk with higher EASIX levels. After adjusting for age, sex, GCS, and CCI in Model 1, the T3 group remained at a significantly higher risk of mortality (HR = 3.28, *p* < 0.0001). Additional adjustments for comorbidities and clinical variables in Model 2 confirmed that the T3 group continued to show a significantly higher mortality risk (HR = 2.68, *p* = 0.01). Trend analysis revealed a significant dose–response relationship, with higher EASIX levels correlating with progressively higher mortality risk across all models (*p* for trend < 0.01) (Table 2).

**Table 2:** Cox proportional hazard ratios (HR) for ACM

Characteristic	Crude model		Model 1		Model 2	
	HR (95%CI)	*P*-value	HR (95%CI)	*P*-value	HR (95%CI)	*P*-value
EASIX (continuous)	1.17(1.09,1.27)	** < 0.0001**	1.19(1.09,1.29)	** < 0.0001**	1.11(1.01,1.23)	** < 0.01**
EASIX category						
T1	Ref		Ref		Ref	
T2	2.19(1.16,4.13)	**0.02**	1.81(0.95,3.45)	0.07	1.89(0.97,3.68)	0.06
T3	3.56(1.98,6.42)	** < 0.0001**	3.28(1.81,5.95)	** < 0.0001**	2.68(1.35,5.34)	** < 0.01**
*P* for trend		** < 0.0001**		** < 0.0001**		**0.004**

Crude model: unadjusted

Model 1: age, sex, GCS, CCI

Model 2: age, sex, GCS, CCI, heart failure, respiratory failure, atrial fibrillation, diabetes, hypertension, hyperlipidemia, renal failure, malignancy, MAP, temperature, SpO2, respiratory rate, heartrate, iv_tPA, thrombectomy

Bold values indicates statistical significance (*p* <0.05).

### Association between log_2_(EASIX) and primary outcomes

**Fig. 3:**
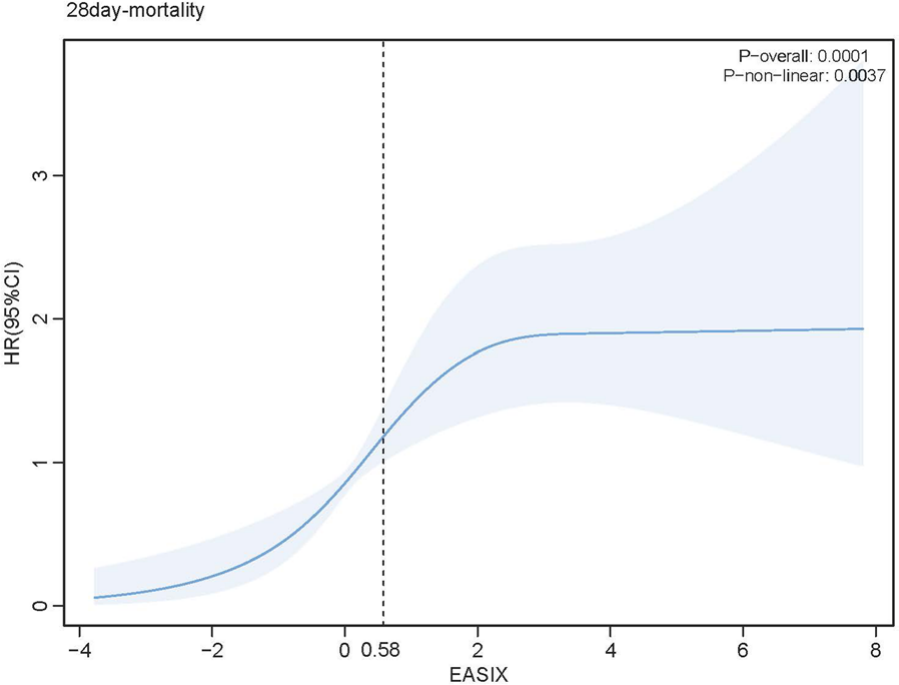
Restricted cubic spline analysis showing the relationship of log_2_ (EASIX) with 28-day ACM

To investigate the nonlinear relationship between log_2_(EASIX) and 28-day ACM, RCS regression analysis was conducted. The results revealed that as log₂(EASIX) increased, the HR for 28-day ACM initially rose, followed by a gradual plateau. Notably, when EASIX surpassed 0.58, the mortality risk increased sharply (Fig. 3).

### Prediction of ACM in SIS using log_2_ (EASIX)

**Fig. 4:**
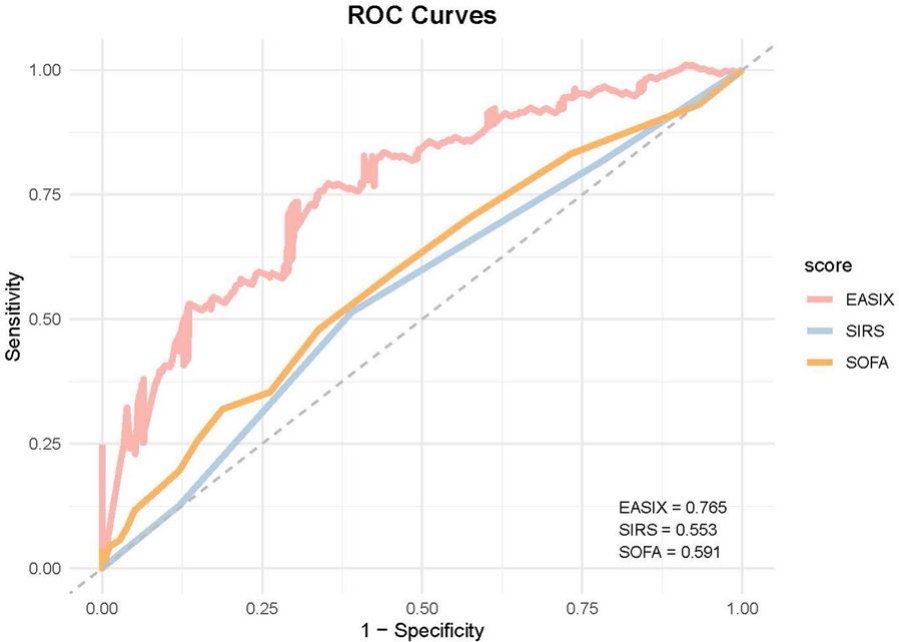
Receiver operating characteristic curves assessing the predictive capability of the log_2_ (EASIX) index for 28-day ACM

To evaluate the predictive performance of log_2_(EASIX) for 28-day ACM, ROC curves were generated for log₂(EASIX), SOFA, and SIRS scores. The results showed that log₂(EASIX) demonstrated superior predictive accuracy for 28-day ACM compared to both SOFA and SIRS, with an area under the curve (AUC) of 0.765, significantly higher than SOFA (0.591) and SIRS (0.553) (Fig. 4).

### Subgroup analysis

**Fig. 5:**
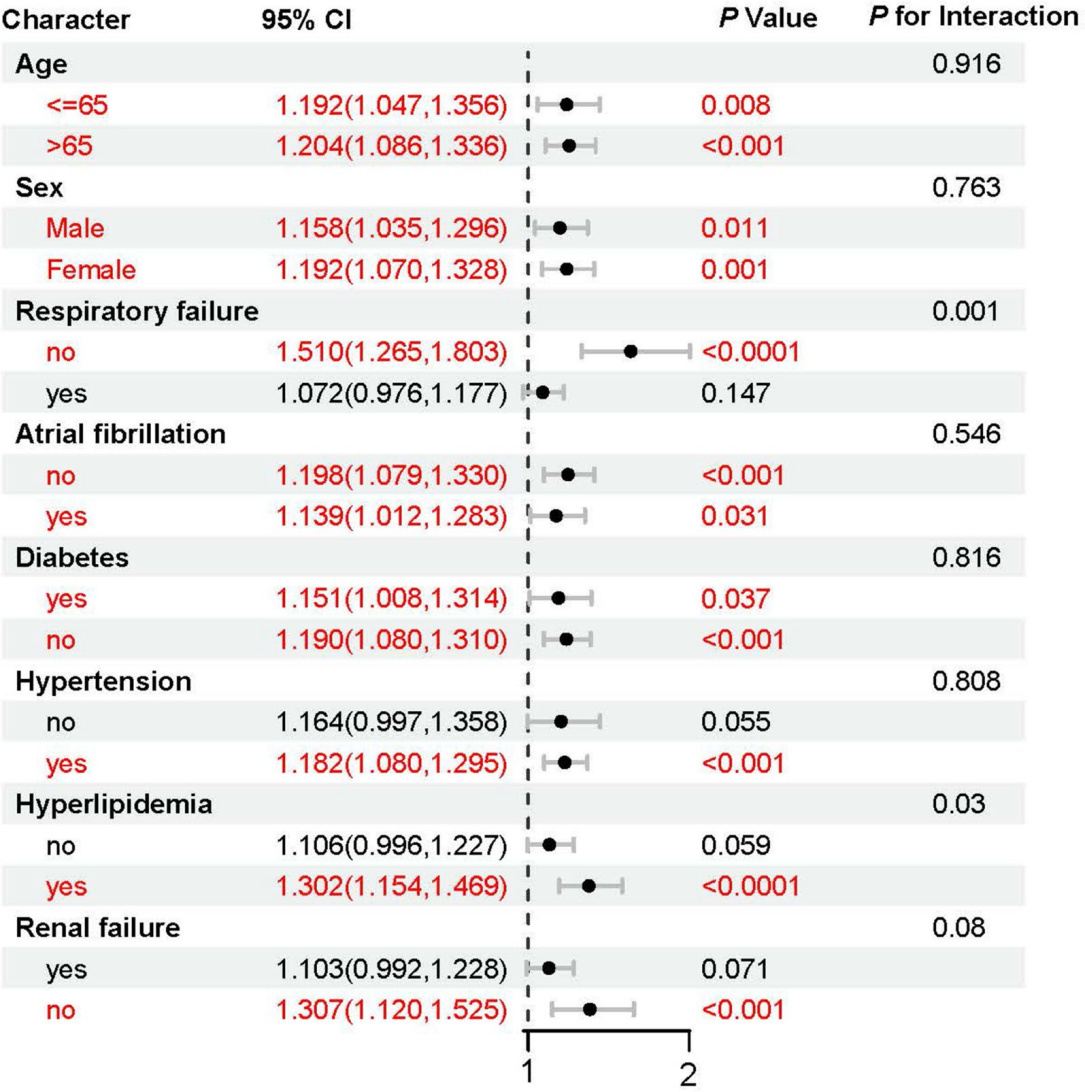
Forest plots of subgroup analyses of the relationship between log2 (EASIX) and 28-ACM in patients with SIS

A subgroup analysis was conducted to further assess the robustness of the association between log_2_(EASIX) and 28-day ACM. In all subgroups, the HRs remained greater than 1, with statistically significant P-values, indicating that elevated EASIX levels were consistently associated with an increased risk of mortality. The interaction *P*-values showed no statistically significant differences, suggesting that the effect of EASIX was consistent across various subgroups, including age, sex, and comorbidities such as atrial fibrillation, diabetes, and hypertension (Fig. 5).

## Discussion

EASIX, derived from LDH, Cr, and PLT, has emerged as a promising prognostic biomarker for predicting mortality risk in critically ill patients. This study, utilizing the MIMIC-IV database, demonstrated that higher EASIX levels are significantly associated with increased 28-day ACM in patients with SIS. Elevated EASIX levels also correlate with prolonged hospital and ICU stays, highlighting its potential for early risk stratification in neurocritical care. Moreover, a nonlinear positive relationship was observed between EASIX and 28-day ACM, with mortality risk rising sharply when EASIX exceeded a threshold of 0.58. Subgroup analyses confirmed the robustness of this association, indicating that EASIX’s predictive power remains consistent across different sex, age, and comorbidity subgroups. EASIX provides crucial early warning information, enabling the identification of high-risk patients and facilitating timely interventions that reduce complications and mortality.

The development of ischemic stroke is driven by complex mechanisms, with endothelial dysfunction playing a key role [17]. Endothelial cells regulate vascular permeability, blood flow, and coagulation, maintaining vascular tone by releasing vasodilators like nitric oxide (NO) and prostacyclin (PGI₂), while also secreting anticoagulant factors to prevent platelet aggregation [18–20]. Pathological conditions such as hypertension, hyperglycemia, and oxidative stress impair endothelial function, leading to reduced NO bioavailability, vasodilation dysfunction, and increased prothrombotic mediators, like thromboxane A₂ [21–23]. These changes exacerbate vasoconstriction, platelet activation, and atherosclerosis, significantly increasing the risk of ischemic stroke [24–26]. Furthermore, acute ischemic stroke triggers a systemic inflammatory response that worsens endothelial dysfunction, promotes vascular remodeling, and contributes to worse outcomes [27, 28]. Ischemic stroke not only disrupts cerebral blood flow, but also activates a systemic inflammatory cascade, exacerbating endothelial dysfunction and accelerating disease progression [29]. This immune response, driven by neuronal injury, spreads to systemic circulation, increasing mortality risk [30]. Acute ischemic stroke induces leukocytosis, elevated C-reactive protein (CRP), and proinflammatory cytokines like tumor necrosis factor-α (TNF-α) and interleukin-6 (IL-6), which further damage the endothelium, increase vascular permeability, and promote thrombosis [31, 32]. These inflammatory mediators also play a critical role in the progression of arterial stenosis, worsening post-stroke pathology [33]. Endothelial dysfunction and thrombosis, accelerated by inflammation, have systemic effects beyond the brain, affecting vital organs like the heart and kidneys, which are particularly vulnerable to ischemia–reperfusion injury [34, 35]. Endothelial injury is linked to coronary microcirculatory dysfunction and impaired coronary perfusion, raising the risk of myocardial infarction and heart failure [36, 37]. Additionally, the inflammatory response triggered by ischemic stroke worsens renal function, especially in patients with pre-existing conditions like diabetes and hypertension, leading to a poorer prognosis [38].

EASIX, a biomarker reflecting endothelial dysfunction, systemic inflammation, and coagulation abnormalities, has demonstrated significant clinical prognostic value. It not only helps clinicians assess patients’ prognostic risks, but also supports early intervention, improving therapeutic outcomes, particularly in critically ill patients [39]. When used in conjunction with existing clinical scoring systems like SOFA and SIRS, EASIX enhances predictive accuracy, allowing for more precise risk stratification and personalized treatment strategies [40]. For example, when SOFA and SIRS alone fail to accurately identify high-risk patients, EASIX serves as a complementary biomarker that provides additional insights to optimize treatment decisions. Furthermore, EASIX’s simplicity and cost-effectiveness make it particularly suitable for resource-limited settings [41]. In such environments, EASIX testing does not require high-cost equipment, offering rapid prognostic assessments that help clinicians quickly identify patients who need urgent intervention. This capability is especially important for improving medical resource efficiency and optimizing the management of acute conditions [42]. EASIX has been validated as a prognostic marker in various diseases, including acute pancreatitis, and hematologic malignancies [43, 44]. Elevated EASIX levels have been shown to correlate with increased mortality risk in these conditions, reinforcing its potential as a widely applicable tool for clinical prognostic assessment.

This study utilized real-world data from the MIMIC-IV database, encompassing a large cohort of SIS patients, thereby enhancing the statistical power and generalizability of the analysis. By employing Cox proportional hazards regression models and adjusting for multiple confounders, the independent association between EASIX and 28-day ACM was robustly assessed. The analysis of EASIX, a simple and cost-effective biomarker, enables the effective identification of high-risk patients and provides timely interventions, thereby reducing complications and mortality, with significant clinical implications and operational feasibility. Nevertheless, this study has certain limitations. Due to its retrospective design, selection bias may have been introduced, limiting the ability to establish causal relationships. Additionally, as the MIMIC-IV database is derived from a single medical center, the findings may not fully represent the broader patient population, thereby limiting their external validity. Future prospective, multicenter studies are required to validate the reliability of EASIX as a prognostic tool for stroke and to assess its applicability across different healthcare settings.

## Conclusion

This study represents a systematic evaluation of the prognostic significance of EASIX in critically ill SIS patients, revealing a significant association between elevated EASIX levels and increased 28-day ACM, as well as prolonged hospital and ICU stays. As a simple and easily accessible biomarker, EASIX holds promise as a valuable tool for early risk stratification in critically ill ischemic stroke patients. However, future multicenter prospective studies are warranted to further establish its clinical utility and elucidate its mechanistic role in the pathogenesis of stroke.

## Data Availability

Publicly available datasets were analyzed in this study. This data can be found here: https://physionet.org/about/database/. For any additional inquiries, please contact the corresponding author(s).
